# The Role of Artificial Intelligence and Machine Learning in Cardiovascular Imaging and Diagnosis: Current Insights and Future Directions

**DOI:** 10.7759/cureus.72311

**Published:** 2024-10-24

**Authors:** Maria Gabriela Cerdas, Sucharitha Pandeti, Likhitha Reddy, Inayat Grewal, Asiya Rawoot, Samia Anis, Jade Todras, Sami Chouihna, Saba Salma, Yuliya Lysak, Saad Ahmed Khan

**Affiliations:** 1 Medicine, Universidad de Ciencias Médicas (UCIMED), San José, CRI; 2 Internal Medicine, Sri Venkateswara Medical College, Tirupati, IND; 3 Internal Medicine, Madras Medical College, Chennai, IND; 4 Radiology, Government Medical College and Hospital, Chandigarh, IND; 5 Internal Medicine, Maharashtra University of Health Sciences, Nashik, IND; 6 Internal Medicine, Dow University of Health Sciences, Karachi, PAK; 7 Biology, Suffolk County Community College, New York, USA; 8 Internal Medicine, University of Toronto, Toronto, CAN; 9 Internal Medicine, Wayne State University Detroit Medical Center, Detroit, USA; 10 Internal Medicine, St. George's University, True Blue, GRD

**Keywords:** artificial intelligence, cardiovascular imaging, computed tomography (ct), diagnostic accuracy, echocardiography, machine learning

## Abstract

Cardiovascular diseases (CVDs) are the major cause of mortality worldwide, emphasizing the critical need for timely and accurate diagnosis. Artificial intelligence (AI) and machine learning (ML) have become revolutionary tools in the healthcare system with significant potential for cardiovascular diagnosis and imaging. AI and ML techniques, including supervised and unsupervised learning, logistic regression, deep learning models, neural networks, and convolutional neural networks (CNNs), have significantly advanced cardiovascular imaging. Applications in echocardiography include left and right ventricular segmentation, ejection fraction measurement, and wall motion analysis. AI and ML hold substantial promise for revolutionizing cardiovascular imaging, demonstrating improvements in diagnostic accuracy and efficiency. This narrative review aims to explore the current applications, advantages, challenges, and future pathways of AI and ML in cardiovascular imaging, highlighting their impact on different imaging modalities and their integration into clinical practice.

## Introduction and background

Heart disease, which is also referred to as cardiovascular disease (CVD), encompasses four conditions, i.e., peripheral artery disease (PAD), cerebrovascular disease, aortic atherosclerosis, and coronary artery disease (CAD), also called coronary heart disease (CHD). These conditions often lead to severe health consequences and a high mortality rate globally [1]. CAD is responsible for approximately 610,000 deaths each year, accounting for roughly 1 in 4 fatalities, making it the leading cause of death in the United States. Globally, it ranks as the third leading cause of mortality, contributing to an estimated 17.8 million deaths annually [2].

The advanced algorithm can be utilized by artificial intelligence (AI) to 'learn' features from an enormous volume of healthcare datasets, which can help improve clinical practice by applying insights gleaned from the process. The accuracy, based on feedback, can also be increased by programming it with self-correcting and learning features. Despite the growing research on AI in the medical domain, the literature mostly focuses on three key illness categories: cancer, CVD, and diseases of the nervous system [3]. Numerous medical domains such as diagnostics, clinical, surgical, prognostic, and rehabilitative techniques can benefit from implementing several AI applications. The two crucial medical areas, diagnosing an illness and clinical decision-making, have also been impacted by AI. Within the realm of AI, machine learning (ML) is the practice of creating or training statistical models using existing real-world data to classify observations or forecast results based on human-provided training [4].

ML involves using algorithms to identify patterns in data, which can be applied to make predictions or enhance understanding of health conditions. In biomedicine, ML is crucial for improving disease diagnostics, enabling earlier and more accurate detection of diseases like cancer and heart disease. By analyzing complex data such as medical records and genetic information, ML helps identify risk factors and optimize personalized treatment plans, ultimately transforming patient care and contributing to more effective healthcare solutions [5].

AI is most urgently needed in biomedicine for disease diagnostics. It enables health professionals to provide earlier and more accurate diagnoses for various diseases [6]. AI can make healthcare more affordable by improving efficiency and reducing costs through automation, early disease detection, and personalized treatments. Automating administrative tasks like billing and scheduling saves time and resources, while AI’s ability to detect diseases early helps avoid costly treatments for advanced conditions. Moreover, AI is frequently used in clinical practice through clinical decision support systems (CDSS) to help clinicians diagnose diseases and make decisions regarding treatment modalities. AI-based CDSSs implement models built on data from patients with comparable cases, whereas conventional CDSSs contrast each patient's characteristics to an existing knowledge base [7].

In the past, data analysis included skilled technicians choosing anatomical structures and taking measurements, which were then reviewed by a cardiologist or radiologist who often provided additional diagnostic information. These measurements were then combined with clinical data to create a unified dataset, and traditional statistical methods were used to assess the relevance of a particular measurement to a clinical result. ML techniques have now advanced not only in registration and segmentation but also in making the measurements typically performed by a human [8].

The average imaging specialist's productivity is challenged by the increasing imaging capabilities and subsequent analyses. The growing number of medical images can be standardized and evaluated using medical AI [9].

Among the most prevalent cardiovascular conditions is CAD, which accounts for one out of every five deaths. Radionuclide myocardial perfusion imaging (MPI) is typically used to diagnose CAD. Incorporating ML to enhance the MPI findings improves personalized risk assessment for patients [10].

Myocardial hypertrophy, heart failure, and myocardial ischemia are some of the diseases in which ML has been used in cardiac magnetic resonance imaging for functional indices, specifically to assess left ventricular function. Many CVDs can be diagnosed and treated using echocardiography; however, the procedure is resource-intensive and needs to be interpreted by a qualified specialist. Echocardiograms often generate complicated data, which is filtered down by the interpreter into information that is clinically meaningful [11]. In two-dimensional (2D) echocardiography, promising results have been displayed by using ML to automate the classification of functional and morphological cardiac characteristics.

## Review

AI and ML in cardiovascular imaging: current insights

ML Techniques in Cardiac Imaging 

AI is a branch of computer science that enables machines, such as computers and robots, to perform tasks that typically require human intelligence. These tasks include pattern recognition, problem-solving, and decision-making. AI systems use algorithms and data to analyze information, learn from it, and make independent decisions without continuous human intervention, mimicking cognitive functions like reasoning and learning [12-15]. ML, a subdivision of AI, operates algorithms for extracting information from available data, improving responses to similar future information by identifying patterns and linking inputs to outputs [10,11,13,16]. Supervised or unsupervised learning can be used to train ML algorithms [10].

Supervised learning is a type of ML where an algorithm is trained on a labeled dataset, meaning that each input is paired with the correct output (or outcome). The algorithm learns to map inputs to outputs, allowing it to make accurate predictions on new, unseen data. Common methods used in supervised learning include CNNs, logistic regression, and support vector machines (SVMs). This approach is widely used in medicine to estimate disease risk, and perform tasks such as regression (predicting continuous outcomes), classification (categorizing data), predictive modeling, and survival analysis [17-23]. In contrast, unsupervised learning works without labeled data, discovering hidden patterns or structures in the data through techniques like clustering analysis and principal component analysis (PCA), and can utilize deep learning to find associations between variables [20-21]. Deep learning is a type of ML that uses neural networks with many layers to automatically learn complex patterns and relationships between variables in large datasets, making it effective for tasks like prediction and pattern recognition [20].

Neural networks, modeled on the structure of human neurons, consist of interconnected nodes organized into input, hidden, and output layers. The hidden nodes process information, while the output layers provide its interpretation, which is needed for advanced data managing and error learning processes [14,16]. Utilizing receiver operating characteristic (ROC) curves, neural networks are evaluated, while the Area Under the Curve (AUC) shows accuracy [11].

CNNs use layers of hidden nodes to process information, thereby improving image recognition and detection accuracy by creating higher-order structural information from meticulous low-level data [22]. CNNs are used for classification, clustering similar images, and object recognition tasks [11].

Deep learning (DL), a subset of ML, applies multilayer neural networks to identify convoluted connections among variables to make predictive representations of patients using health records in an unsupervised manner. An advantage of DL is that it requires less data preprocessing compared to other methods and learns features from raw data. Important applications in the medical field include identifying and segmenting objects in digital images or videos for classification [21].

Applications in various imaging techniques 

Echocardiography

The most extensively utilized cardiology imaging method is echocardiography because it provides multiple benefits such as portability, accessibility, and affordability [9,10,19]. Nonetheless, precise interpretation depends on medical expertise and image quality, which might end up affecting clinical outcomes [19]. AI and ML can be used to improve accuracy and efficiency while minimizing the dependence on operator skill, therefore facilitating early diagnosis and improving cardiac disease management through prompt intervention (Table 1) [10].

AI rapidly analyzes cardiac images and accurately measures chamber dimensions, wall thickness, volumes, and cardiac structures [19,22]. DL has greatly enhanced the precision and accuracy of cardiovascular imaging segmentation. It utilizes multi-scale deep reinforcement learning to identify landmarks and delineate heart and vascular structures [20].

Another use of AI is creating knowledge-based reconstructions for measuring right ventricle volume, which is difficult to assess through 2D imaging [22]. Medvedofsky et al. determined that automated 3D echocardiography offers an accurate and reproducible alternative to traditional manual methods with the potential to facilitate the full integration of this technique into clinical practice [24]. In 2016 Narula et al. explored the potential for diagnosis of a ML system utilizing speckle-tracking echocardiographic data to automatically distinguish physiological hypertrophy from hypertrophic cardiomyopathy observed among athletes. The study concluded that these algorithms can effectively contribute to distinguishing these two patterns [25].

Currently, ejection fraction calculation involves manually detecting the end-diastolic volume and left ventricular end-systolic volume, which can vary significantly among observers. For this measurement, 3D echocardiography is now preferred, however, it has its limitations in clinical practice. ML can be used to automate this process, offering a more precise volume measurement and therefore a precise estimated ejection fraction [18,22]. Knackstedt et al. concluded that fully automated measurement of EF is achievable in a few seconds and matches the accuracy of manual tracking [26].

Echocardiography wall motion anomalies might differ in interpretation as they rely on the experience of the observer and, hence can vary between individuals. An objective approach is provided by using a deep learning algorithm to evaluate regional wall motion abnormalities (RWMAs) [15,23]. The study conducted by Kusunose et al. showed that the AUC produced by the deep learning algorithm was greater than the resident readers' results (0.99 vs 0.90) and equivalent to those of cardiologists and sonographer readers (0.99 vs 0.98) [23].

These algorithms can also evaluate the severity of heart valve diseases (valvulopathies) by analyzing echocardiographic data, providing an objective assessment that helps clinicians make more accurate decisions during preoperative evaluations [19]. Samad et al. published a study to demonstrate the significance of ML models in predicting crucial clinical outcomes. These models outperformed standard linear regression methods and the Framingham risk score currently used to assess the patient’s risk of mortality [27].

Computed Tomography

A key diagnostic equipment for various medical conditions is computed tomography which provides a detailed view of the coronary artery anatomy, including the detection of plaques, stenosis, coronary calcification, and scoring [9,28,29]. AI image enhancement can reduce the number of image acquisitions, radiation, and contrast doses. Deep learning algorithms can convert non-contrast CCT scans into contrast ones, aiding in the identification of coronary artery calcification [16].

ML algorithms in cardiovascular computed tomography enable accurate detection and segmentation of plaques (Table 1). A ML-based algorithm was built by Kang et al. to identify coronary arterial lesions from computed tomography angiography (CTA), achieving 93% sensitivity and 95% specificity surpassing the accuracy of three experienced expert readers. The study showed that, with an AUC of 0.94, this algorithm could distinguish between non-obstructive lesions (<50% stenosis) and obstructive (≥50% stenosis) [17].

**Table 1:** Description of multiple research studies demonstrating the application of machine learning in echocardiography, computed tomography, and MRI ML: Machine learning; AUC: Area under the curve; DL: Deep learning.

Study	Type of Imaging	Study's Results
Medvedofsky et al. [24]	Echocardiography	Automated 3D echocardiography offers an accurate and reproducible alternative to traditional manual methods.
Narula et al. [25]	Echocardiography	Data from speckle-tracking echocardiography can distinguish between athletes' physiological hypertrophy and hypertrophic cardiomyopathy.
Knackstedt et al. [26]	Echocardiography	Automated measurement of ejection fraction is achievable in a few seconds and matches the accuracy of manual tracking.
Kusunose et al. [23]	Echocardiography	The AUC generated by the DL algorithm was greater than the results of the resident readers (0.99 vs 0.90) and similar to those of cardiologists and sonographer readers (0.99 vs 0.98).
Samad et al. [27]	Echocardiography	ML models outperformed standard linear regression methods and the Framingham risk score.
Kang et al. [17]	Computed Tomography	An ML-based algorithm could identify both non-obstructive and obstructive lesions, achieving an AUC of 0.94 (93% sensitivity and 95% specificity).
Han et al. [19]	Computed Tomography	ML algorithm results to predict rapid coronary plaque progression (AUC of 0.83) were superior to the atherosclerotic cardiovascular disease risk score (AUC of 0.60) and the Duke coronary artery disease score (AUC of 0.74).
Zreik et al. [30]	Computed Tomography	DL methods can effectively identify stenosis, decreasing the need for invasive fractional flow reserve measurements.
Nakanishi et al. [31]	Computed Tomography	The combined approach of ML models and non-contrast CT imaging showed increased AUC compared to ASCVD and CAC scores alone (0.826 vs. 0.821 and 0.804 vs. 0.781 respectively).
Lin et al. [32]	MRI	Left ventricular scar tissue can be distinguished from healthy myocardium by DL convolutional neural networks.
Fahmy et al. [33]	MRI	Automated myocardial alignment, segmentation, and calculation of T1 for myocardial T1 mapping were able to reduce the need for manual measurements and provided results comparable to those obtained by expert readers.

Han et al. investigated the ML use to anticipate rapid coronary plaque progression. They found that the most crucial feature for predicting plaque progression was the CT-based plaque features, followed by qualitative CT-based features, and lastly by clinical and laboratory findings. The results of the study (AUC of 0.83) established its advantages over the atherosclerotic CVD risk (AUC of 0.60) and Duke coronary artery disease score (AUC of 0.74) [19].

Coronary computed tomography (CCT) angiography is the preferred method for diagnosing coronary artery disease because of its negative predictive value and high sensitivity for detecting coronary stenosis [28]. Zreik et al. introduced an algorithm that uses automatic analysis of the left ventricle from a single resting coronary CT angiography scan for detecting patients with substantial coronary artery stenosis. They demonstrated that deep learning methods can effectively identify stenosis, potentially reducing the need for invasive fractional flow reserve measurements [30]. There is a study done by Nakanishi et al. reported that deaths from CHD and CVD can be better predicted using ML models that use clinical and non-contrast CT imaging data, making them superior to traditional scores. The combined approach showed increased AUC than atherosclerotic cardiovascular disease (ASCVD) and Coronary Artery Calcium (CAC) scores alone (0.826 vs 0.821 and 0.804 vs 0.781, respectively) [31].

MRI

AI, particularly ML and DL have been incorporated into cardiac MRI for various aspects of cardiovascular diagnostics, such as functional assessment, tissue characterization, and fibrosis detection [28]. MRI has set the benchmark in evaluating ejection fraction, left ventricular volume, cardiac structure, and systolic function [10]. Despite the standardization and image speed of image acquisition, the analysis process is lengthy and operator dependent leading to inconsistencies. DL can automate this process with high accuracy and precision [32]. 

AI improves cardiac MRI diagnostics by enabling and facilitating detailed tissue characterization. Methods including gadolinium enhancement imaging, T1 and T2 mapping, and diffusion-weighted imaging are essential for identifying myocardial injuries, ischemic scars, and fibrotic areas [9,31]. Native T1 mapping modalities are effective in detecting raised extracellular compartments, while T2 mapping is highly precise in evaluating myocardial edema. Patients with ischemic or hypertrophic cardiomyopathies can have accurate scar volume measurements because deep learning CNNs can differentiate between healthy myocardium and left ventricular scar tissue [33].

Cardiovascular magnetic resonance myocardial native T1 mapping assesses diffuse interstitial fibrosis in the heart [34]. For myocardial T1 mapping, Fahmy et al. developed an automated method that merges myocardial alignment, segmentation, and calculation of T1. The proposed technique employed a fully convolutional network to automatically segment the myocardium, therefore reducing the need for manually measuring and providing rapid and consistent parameters that are comparable to those obtained by expert readers [34].

Advantages and challenges 

Table 2 summarizes the benefits and challenges associated with the integration of AI in cardiovascular imaging. While AI offers significant potential in improving diagnostic accuracy, operational efficiency, and early detection of high-risk patients, it also faces challenges such as the need for substantial training, potential biases, and issues with reproducibility and standardization [9,10,28,29,35,36].

**Table 2:** Advantages and challenges of AI in clinical practice. AI: Artificial intelligence.

Advantages	Challenges
AI in cardiovascular imaging advances pharmacological treatments and early illness detection [9,10,35].	AI requires substantial training and numerous iterations before use [28].
Automates tasks, allowing physicians to focus on patient care and education [10].	Accurate AI models need large, well-labeled data sets, which are scarce and labor-intensive [36].
Enhances diagnostic accuracy and individualized health treatment by combining imaging data with clinical features and biomarkers [9,35].	Integration of AI in medical imaging is hindered by varying diagnostic criteria and privacy concerns [36].
Identifies high-risk individuals for heart failure with 85% accuracy, reducing unnecessary hospitalizations [35].	Machine Learning (ML) can overfit training data and underfit new data [29].
Improves detection of subtle patterns and anomalies in medical imaging, reducing human errors and maintaining consistency [37].	Deep Learning operates as a "black box," causing unexpected biases and ambiguous recommendations [28,29].
Enhances operational efficiency by accelerating image reading, matching expert radiologist sensitivity, and shortening evaluation times [37].	AI can introduce biases if clinicians over-rely on computerized diagnosis outcomes without thorough examination [10].
AI-powered predictive analytics allow for early detection and intervention of health risks [37].	Issues with reproducibility and standardization in AI models make it difficult to compare outcomes across different suppliers [10].
-	AI faces challenges in drawing generalized conclusions across diverse populations due to variations in social, racial, and socioeconomic factors, which can affect the accuracy and applicability of predictive models [38].

Case studies and recent developments

The incorporation of AI in cardiac imaging has led to substantial advancements in diagnosing and managing CVDs. AI has enhanced the accuracy, efficiency, and reproducibility of imaging techniques like echocardiography, MRI, and computed tomography (CT).

Echocardiography

Echocardiography is crucial in cardiovascular medicine for assessing left ventricular (LV) function. Recent advancements have introduced AI models for automatic detection of cardiac anomalies, improving diagnostic accuracy and reducing operator variability. A notable development is AI for left ventricular segmentation, which has demonstrated strong concordance with human expert readings, particularly in measuring LV volumes and ejection fraction (EF). These advancements improve diagnostic precision and allow for large-scale retrospective analyses by minimizing inter-operator variability [38,39].

Computed Tomography

AI-assisted coronary CT angiography has gained prominence in computed tomography. CNNs are employed for detecting and quantifying atherosclerotic plaques, providing a detailed characterization of coronary artery disease that is both accurate and efficient. This application of AI not only enhances diagnostic capabilities but also aids in cardiovascular risk stratification by offering precise measurements of plaque burden and composition [40,41].

MRI

MRI has also benefited from AI innovations, particularly in functional MRI for detailed tissue analysis. AI-driven models have been developed for detecting myocardial fibrosis, significantly enhancing the accuracy of prognostic evaluations. These models enable more detailed tissue analysis, which is critical for managing conditions like cardiomyopathies and ischemic heart disease [33,39,42].

Future directions 

Incorporating AI training into medical education enhances diagnostics and learning efficiency. Medical professionals need foundational AI understanding for better integration and adaptability to new technologies. Despite growing demand, many lack AI knowledge, making awareness of training programs crucial. Courses like those from Harvard and MIT explore AI applications in healthcare, aiding future readiness [43].

The development of advanced AI models and multimodal medical imaging data integration will revolutionize cardiovascular imaging and diagnostics. Since 2020, studies on AI and ML in this field have surged, reflecting growing interest [44]. Future AI models will be more adaptable and efficient, excelling in tasks like segmentation, classification, and prediction. Deep learning advancements have already improved automation in these areas [44,45]. Incorporating data from various imaging techniques, such as echocardiography and MRI, promises more accurate diagnoses and better treatment planning. This comprehensive approach will enhance clinical decision-making and patient outcomes [46].

AI in cardiovascular imaging must be accurate, robust, and transparent. Key concerns include consent, rights, robustness, usability, bias, privacy, transparency, explainability, and accountability. Trustworthy AI must meet requirements such as human oversight, technical safety, privacy, transparency, fairness, and accountability. To address bias, AI should ensure fairness for individuals and groups, involving multidisciplinary teams, defining fairness contextually, standardizing key variables, and probing data for imbalances. Ensuring patient data privacy and security is crucial, requiring transparent data collection, multi-center data, statistical fairness methods, and cybersecurity measures [47,48].

AI and ML can enhance personalized medicine by analyzing large data sets, including clinical, imaging, demographic, and genetic information, to provide individualized diagnoses and treatments [28]. They enable pattern recognition in multi-dimensional data and support personalized care through cloud-based infrastructures [49]. AI also improves clinical trials by analyzing preliminary results, aiding disease classification, optimizing enrollment strategies, and enhancing randomization [50].

While this review focuses on the technical advancements of AI in cardiovascular imaging, future reviews could explore the ethical conundrums AI presents in medicine, including algorithmic bias, data privacy, and its impact on healthcare disparities.

## Conclusions

Diagnostic imaging has advanced significantly with the merger of ML and AI, leading to improved detection and management of CVDs. Numerous studies have shown AI's ability to enhance the precision, speed, and accuracy of various imaging processes, including echocardiography, MRI, and computed tomography. AI application in cardiology facilitates tasks such as ventricular segmentation, ejection fraction measurement, wall motion analysis, plaque quantification, stenosis detection, coronary artery analysis, tissue characterization, and fibrosis detection. Although AI has outperformed conventional techniques in certain areas, it still has several limitations, including the requirement for extensive, well-labeled datasets, potential model overfitting, and issues related to transparency and bias. Therefore, AI should be utilized as a complementary tool rather than a replacement for clinical decision-making. Despite these challenges, the ongoing research on AI promises more effective and personalized cardiovascular healthcare as technology continues to improve.

## References

[1] Olvera LE, Ballard BD, Jan A (2024). Cardiovascular Disease. https://www.ncbi.nlm.nih.gov/books/NBK535419/.

[2] Brown JC, Gerhardt TE, Kwon E (2024). Risk factors for coronary artery disease. https://pubmed.ncbi.nlm.nih.gov/32119297/#:~:text=Excerpt,with%2017.8%20million%20deaths%20annually.

[3] Jiang F, Jiang Y, Zhi H (2017). Artificial intelligence in healthcare: past, present and future. Stroke Vasc Neurol.

[4] Singh S, Kumar R, Payra S, Singh SK (2023). Artificial intelligence and machine learning in pharmacological research: bridging the gap between data and drug discovery. Cureus.

[5] Wiens J, Shenoy ES (2018). Machine learning for healthcare: on the verge of a major shift in healthcare epidemiology. Clin Infect Dis.

[6] Rong G, Mendez A, Bou Assi E (2020). Artificial intelligence in healthcare: review and prediction case studies. Engineering.

[7] Amann J, Blasimme A, Vayena E, Frey D, Madai VI (2020). Explainability for artificial intelligence in healthcare: a multidisciplinary perspective. BMC Med Inform Decis Mak.

[8] Sermesant M, Delingette H, Cochet H, Jaïs P, Ayache N (2021). Applications of artificial intelligence in cardiovascular imaging. Nat Rev Cardiol.

[9] Siegersma KR, Leiner T, Chew DP, Appelman Y, Hofstra L, Verjans JW (2019). Artificial intelligence in cardiovascular imaging: state of the art and implications for the imaging cardiologist. Neth Heart J.

[10] Patel B, Makaryus AN (2022). Artificial intelligence advances in the world of cardiovascular imaging. Healthcare (Basel).

[11] Haq IU, Haq I, Xu B (2021). Artificial intelligence in personalized cardiovascular medicine and cardiovascular imaging. Cardiovasc Diagn Ther.

[12] Dey D, Slomka PJ, Leeson P, Comaniciu D, Shrestha S, Sengupta PP, Marwick TH (2019). Artificial intelligence in cardiovascular imaging: JACC state-of-the-art review. J Am Coll Cardiol.

[13] Lin A, Kolossváry M, Motwani M, Išgum I, Maurovich-Horvat P, Slomka PJ, Dey D (2021). Artificial intelligence in cardiovascular imaging for risk stratification in coronary artery disease. Radiol Cardiothorac Imaging.

[14] Oikonomou EK, Siddique M, Antoniades C (2020). Artificial intelligence in medical imaging: a radiomic guide to precision phenotyping of cardiovascular disease. Cardiovasc Res.

[15] Yoon YE, Kim S, Chang HJ (2021). Artificial intelligence and echocardiography. J Cardiovasc Imaging.

[16] Badano LP, Keller DM, Muraru D, Torlasco C, Parati G (2020). Artificial intelligence and cardiovascular imaging: a win-win combination. Anatol J Cardiol.

[17] Kang D, Dey D, Slomka PJ (2015). Structured learning algorithm for detection of nonobstructive and obstructive coronary plaque lesions from computed tomography angiography. J Med Imaging (Bellingham).

[18] Ben Ali W, Pesaranghader A, Avram R (2021). Implementing machine learning in interventional cardiology: the benefits are worth the trouble. Front Cardiovasc Med.

[19] Han D, Kolli KK, Al'Aref SJ (2020). Machine learning framework to identify individuals at risk of rapid progression of coronary atherosclerosis: From the PARADIGM registry. J Am Heart Assoc.

[20] Al'Aref SJ, Anchouche K, Singh G (2019). Clinical applications of machine learning in cardiovascular disease and its relevance to cardiac imaging. Eur Heart J.

[21] Johnson KW, Torres SJ, Glicksberg BS (2018). Artificial intelligence in cardiology. J Am Coll Cardiol.

[22] Mathur P, Srivastava S, Xu X, Mehta JL (2020). Artificial intelligence, machine learning, and cardiovascular disease. Clin Med Insights Cardiol.

[23] Kusunose K, Abe T, Haga A, Fukuda D, Yamada H, Harada M, Sata M (2020). A deep learning approach for assessment of regional wall motion abnormality from echocardiographic images. JACC Cardiovasc Imaging.

[24] Medvedofsky D, Mor-Avi V, Amzulescu M (2018). Three-dimensional echocardiographic quantification of the left-heart chambers using an automated adaptive analytics algorithm: multicentre validation study. Eur Heart J Cardiovasc Imaging.

[25] Narula S, Shameer K, Salem Omar AM, Dudley JT, Sengupta PP (2016). Machine-learning algorithms to automate morphological and functional assessments in 2D echocardiography. J Am Coll Cardiol.

[26] Knackstedt C, Bekkers SC, Schummers G (2015). Fully automated versus standard tracking of left ventricular ejection fraction and longitudinal strain: the FAST-EFs multicenter study. J Am Coll Cardiol.

[27] Samad MD, Ulloa A, Wehner GJ (2019). Predicting survival from large echocardiography and electronic health record datasets: optimization with machine learning. JACC Cardiovasc Imaging.

[28] Seetharam K, Min JK (2020). Artificial intelligence and machine learning in cardiovascular imaging. Methodist Debakey Cardiovasc J.

[29] Laad M, Kotecha K, Patil K, Pise R (2022). Cardiac diagnosis with machine learning: a paradigm shift in cardiac care. Appl Artif Intell.

[30] Zreik M, Lessmann N, van Hamersvelt RW (2018). Deep learning analysis of the myocardium in coronary CT angiography for identification of patients with functionally significant coronary artery stenosis. Med Image Anal.

[31] Nakanishi R, Slomka PJ, Rios R (2021). Machine learning adds to clinical and CAC assessments in predicting 10-year CHD and CVD deaths. JACC Cardiovasc Imaging.

[32] Lanzafame LR, Bucolo GM, Muscogiuri G (2023). Artificial intelligence in cardiovascular CT and MR imaging. Life (Basel).

[33] Lin A, Pieszko K, Park C, Ignor K, Williams MC, Slomka P, Dey D (2023). Artificial intelligence in cardiovascular imaging: enhancing image analysis and risk stratification. BJR Open.

[34] Fahmy AS, El-Rewaidy H, Nezafat M, Nakamori S, Nezafat R (2019). Automated analysis of cardiovascular magnetic resonance myocardial native T(1) mapping images using fully convolutional neural networks. J Cardiovasc Magn Reson.

[35] Nitisha TS (2022). The use of AI in advanced medical imaging. J Posit Sch Psychol.

[36] Liao J, Huang L, Qu M, Chen B, Wang G (2022). Artificial intelligence in coronary CT angiography: current status and future prospects. Front Cardiovasc Med.

[37] Khalifa M, Albadawy M (2024). AI in diagnostic imaging: revolutionising accuracy and efficiency. Comput Methods Programs Biomed Update.

[38] Borkowski P, Borkowska N, Mangeshkar S, Adal BH, Singh N (2024). Racial and socioeconomic determinants of cardiovascular health: a comprehensive review. Cureus.

[39] Huang S, Zhao T, Liu C (2021). Portable device improves the detection of atrial fibrillation after ablation. Int Heart J.

[40] Kerndt CC, Chopra R, Weber P, Rechenberg A, Summers D, Boyden T, Langholz D (2023). Using artificial intelligence to semi-quantitate coronary calcium as an 'incidentaloma' on non-gated, non-contrast CT scans, a single-center descriptive study in West Michigan. Spartan Med Res J.

[41] Rim TH, Lee CJ, Tham YC (2021). Deep-learning-based cardiovascular risk stratification using coronary artery calcium scores predicted from retinal photographs. Lancet Digit Health.

[42] Boyd C, Brown G, Kleinig T, Dawson J, McDonnell MD, Jenkinson M, Bezak E (2021). Machine learning quantitation of cardiovascular and cerebrovascular disease: a systematic review of clinical applications. Diagnostics (Basel).

[43] Bowman L, Baras A, Bombien R (2020). Understanding the use of observational and randomized data in cardiovascular medicine. Eur Heart J.

[44] Paranjape K, Schinkel M, Nannan Panday R, Car J, Nanayakkara P (2019). Introducing artificial intelligence training in medical education. JMIR Med Educ.

[45] Rouzrokh P, Khosravi B, Vahdati S (2023). Machine learning in cardiovascular imaging: a scoping review of published literature. Curr Radiol Rep.

[46] Antoniades C, Oikonomou EK (2021). Artificial intelligence in cardiovascular imaging-principles, expectations, and limitations. Eur Heart J.

[47] Marey A, Serdysnki KC, Killeen BD, Unberath M, Umair M (2024). Applications and implementation of generative artificial intelligence in cardiovascular imaging with a focus on ethical and legal considerations: what cardiovascular imagers need to know!. BJR Artificial Intell.

[48] van der Veer SN, Riste L, Cheraghi-Sohi S (2021). Trading off accuracy and explainability in AI decision-making: findings from 2 citizens' juries. J Am Med Inform Assoc.

[49] Wellnhofer E (2022). Real-world and regulatory perspectives of artificial intelligence in cardiovascular imaging. Front Cardiovasc Med.

[50] Krittanawong C, Johnson KW, Tang WW (2019). How artificial intelligence could redefine clinical trials in cardiovascular medicine: lessons learned from oncology. Per Med.

